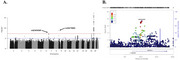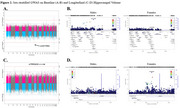# Sex‐specific genetic predictors of hippocampal volume in older adults

**DOI:** 10.1002/alz70856_104200

**Published:** 2025-12-26

**Authors:** Skylar Walters, Derek B. Archer, Kwangsik Nho, Andrew J. Saykin, Christos Davatzikos, Guray Erus, Shannon L Risacher, Mohamad Habes, Di Wang, Reisa A. Sperling, Mohamad J. Alshikho, Adam Brickman, Marilyn S. S. Albert, Anja Soldan, Yang An, Lori L Beason‐Held, Murat Bilgel, Susan M. Resnick, Toshiko Tanaka, Keenan A. Walker, Sarah Biber, Walter W. Kukull, Lisa L. Barnes, David A. A. Bennett, Philip L. De Jager, Vilas Menon, Julie A Schneider, Corinne D. Engelman, Jason M Fletcher, Sterling C Johnson, Qiongshi Lu, Jie Song, L. Taylor Davis, Mary Ellen I. Koran, Dandan Liu, Kimberly R. Pechman, Niranjana Shashikumar, Angela L. Jefferson, Timothy J. Hohman, Logan Dumitrescu, The Alzheimer's Disease Neuroimaging Initiative (ADNI); The Alzheimer's Disease Sequencing Project (ADSP)

**Affiliations:** ^1^ Vanderbilt Memory & Alzheimer's Center, Vanderbilt University Medical Center, Nashville, TN, USA; ^2^ Department of Radiology and Imaging Sciences, Indiana Alzheimer's Disease Research Center, Center for Neuroimaging, Indiana University School of Medicine, Indianapolis, IN, USA; ^3^ Department of Medical and Molecular Genetics, Indiana University School of Medicine, Indianapolis, IN, USA; ^4^ Department of Radiology and Imaging Sciences, Indiana University School of Medicine, Indianapolis, IN, USA; ^5^ Department of Radiology, University of Pennsylvania, Philadelphia, PA, USA; ^6^ University of Pennsylvania, Philadelphia, PA, USA; ^7^ Glenn Biggs Institute for Alzheimer's & Neurodegenerative Diseases, University of Texas Health Sciences Center at San Antonio, San Antonio, TX, USA; ^8^ UT Health San Antonio, San Antonio, TX, USA; ^9^ Massachusetts General Hospital, Harvard Medical School, Boston, MA, USA; ^10^ Taub Institute for Research on Alzheimer's Disease and the Aging Brain, New York, NY, USA; ^11^ Taub Institute for Research on Alzheimer's Disease and the Aging Brain, Vagelos College of Physicians and Surgeons, Columbia University, New York, NY, USA; ^12^ Department of Neurology, The Johns Hopkins University School of Medicine, Baltimore, MD, USA; ^13^ Department of Neurology, Johns Hopkins University School of Medicine, Baltimore, MD, USA; ^14^ Laboratory of Behavioral Neuroscience, National Institute on Aging Intramural Research Program, National Institutes of Health, Baltimore, MD, USA; ^15^ Laboratory of Behavioral Neuroscience, National Institute on Aging, Baltimore, MD, USA; ^16^ Laboratory of Behavioral Neurosciences, National Institute on Aging Intramural Research Program, National Institutes of Health, Baltimore, MD, USA; ^17^ National Institute on Aging, Baltimore, MD, USA; ^18^ Laboratory of Behavioral Neuroscience, National Institute on Aging, Intramural Research Program, Baltimore, MD, USA; ^19^ University of Washington, Seattle, WA, USA; ^20^ Department of Epidemiology, School of Public Health, University of Washington, Seattle, WA, USA; ^21^ Rush Alzheimer's Disease Center, Rush University Medical Center, Chicago, IL, USA; ^22^ Center for Translational & Computational Neuroimmunology, Columbia University Irving Medical Center, New York, NY, USA; ^23^ Department of Population Health Sciences, University of Wisconsin School of Medicine and Public Health, Madison, WI, USA; ^24^ La Follette School of Public Affairs, University of Wisconsin‐Madison, Madison, WI, USA; ^25^ Alzheimer's Disease Research Center, University of Wisconsin‐Madison, Madison, WI, USA; ^26^ Wisconsin Alzheimer's Disease Research Center, University of Wisconsin School of Medicine and Public Health, Madison, WI, USA; ^27^ Department of Biostatistics and Medical Informatics, University of Wisconsin‐Madison, Madison, WI, USA; ^28^ Department of Statistics, University of Wisconsin, Madison, WI, USA; ^29^ Department of Neurology, Vanderbilt Memory & Alzheimer's Center, Vanderbilt University Medical Center, Nashville, TN, USA; ^30^ Vanderbilt Memory and Alzheimer's Center, Vanderbilt University School of Medicine, Nashville, TN, USA

## Abstract

**Background:**

Hippocampal volume is an important *in vivo* imaging marker for Alzheimer's disease (AD) risk and progression. Reported sex differences show accelerated hippocampal atrophy in females compared to males as AD‐related pathology increases. However, genome‐wide association studies (GWAS) of hippocampal volume have been mainly conducted in mid‐life participants with no AD pathology and have not been systemically examined for sex‐specific genetic effects. We investigated the sex‐specific genetic architecture of hippocampal volume in eight aging and AD cohorts.

**Method:**

This study included 5,523 non‐Hispanic White participants (N_Males_=2,549; N_Females_=2,974; mean baseline age=72 yrs; mean number of visits=2.3; 32.8% cognitively impaired). Hippocampal volume and estimated total intracranial volume (eTIV) were segmented from T_1_‐weighted MRIs using Hippodeep. Total hippocampal volume (Left+Right) and eTIV were harmonized using *neuroCombat* in R. Longitudinal slopes for hippocampal volume were calculated with linear mixed‐effects models. GWAS were performed, at baseline and longitudinally, by cohort in all participants, sex‐stratified, and sex‐interaction models. Models covaried for baseline age, sex (in all participants), eTIV, and the first five genetic principal components. Longitudinal models also covaried for diagnosis conversion over time. Results were meta‐analyzed across cohorts.

**Results:**

We identified three genome‐wide significant genetic loci associated with hippocampal volume. Specifically, a chromosome 6 locus (index SNP rs62434269; MAF=0.33) near *ARID1B* was associated at baseline in all participants (β=0.10, *p* = 3.54x10^‐8^) (Figure 1). Further, we found a locus on chromosome 8 (rs34173062; MAF=0.09), which is a previously reported AD risk variant and eQTL for *SHARPIN* (β=‐0.22, *p* = 1.68E‐09), with significant effects in males (β_Males_=‐0.32, p_Males_=2.14x10^‐9^) but not females (β_Females_=‐0.11, p_Females_=0.03; p_sex‐interaction_=0.004) (Figure 2A‐B). A chromosome 14 locus (rs75592630; MAF=0.05) near *AKAP6*, a gene previously associated with cognition, was associated with hippocampal volume change over time in females (β_Females_=‐0.28, p_Females_=1.19x10^‐9^) but not males (β_Males_=‐0.01, p_Males_=0.79; p_sex‐interaction_=0.003) (Figure 2C‐D).

**Conclusion:**

We extend findings of a previously reported AD risk variant near *SHARPIN* to hippocampal volume and provide evidence of novel sex‐specific genetic effects. While replication is warranted, our results suggest the importance of genetic predictors and sex differences on imaging biomarkers.